# *Borrelia miyamotoi* in Saint Petersburg and Leningrad Oblast, Russia: A Multi-Level Assessment of Ticks, Rodents, and Human Exposure

**DOI:** 10.3390/tropicalmed11060166

**Published:** 2026-06-18

**Authors:** Ivan Lyzenko, Olga Stukolova, Nikolay Tokarevich, Marina Sokolova, Regina Baimova, Islam Karmokov, Ekaterina Riabiko, Daria Grechishkina, Gelena Lunina, Vladimir Dedkov

**Affiliations:** 1 Saint Petersburg Pasteur Institute, 197101 St. Petersburg, Russia; tokarevich@pasteurorg.ru (N.T.); baimova@pasteurorg.ru (R.B.); karmokov@pasteurorg.ru (I.K.); riabiko@pasteurorg.ru (E.R.); grechishkina@pasteurorg.ru (D.G.); lunina@pasteurorg.ru (G.L.); vgdedkov@yandex.ru (V.D.); 2 Central Research Institute of Epidemiology, 111123 Moscow, Russia; ovasika@yandex.ru (O.S.); m.sokolova@cmd.su (M.S.); 3 Martsinovsky Institute of Medical Parasitology, Tropical and Vector Borne Diseases, Sechenov First Moscow State Medical University, 119435 Moscow, Russia

**Keywords:** *Borrelia miyamotoi*, ixodid ticks, *Ixodes ricinus*, *Ixodes persulcatus*, small mammals, reservoir hosts, seroprevalence, natural foci, Leningrad Oblast, northwestern Russia, tick-borne infections

## Abstract

**Background:** *Borrelia miyamotoi* is an emerging tick-borne pathogen causing relapsing fever in humans. Although Saint Petersburg and the surrounding Leningrad Oblast harbor a high abundance of ixodid ticks (*I. ricinus*, *I. persulcatus*), no integrated assessment has yet simultaneously addressed pathogen circulation in vectors, reservoir hosts, and human populations in this specific northwestern region of Russia. **Methods**: During 2022–2024, we collected 1518 questing adult ticks and trapped 516 small mammals in Saint Petersburg and Leningrad Oblast. *B. miyamotoi* DNA was detected by real-time PCR. Sera from 3743 randomly selected volunteers (1553 from Saint Petersburg, 2190 from Leningrad Oblast) were tested for anti-*B. miyamotoi* IgG/IgM using a protein microarray (antigens: GlpQ, Vmps, flagellin). Infection rates and seroprevalence with 95% Wilson confidence intervals were compared using chi-square tests. **Results**: The overall tick infection rate was 3.78% (57/1506). *I. ricinus* had a significantly higher prevalence (4.94%; 95% CI: 3.67–6.60%) than *I. persulcatus* (2.29%; 95% CI: 1.39–3.74%; *p* = 0.011). Ticks from Leningrad Oblast also showed markedly elevated infection rates (4.98%; 95% CI: 3.75–6.58%) compared to those from Saint Petersburg (1.89%; 95% CI: 1.06–3.35%; *p* = 0.004). Small mammals exhibited substantially higher infection rates in Leningrad Oblast (39.44%; 95% CI: 31.78–47.65%) than in Saint Petersburg (13.90%; 95% CI: 10.76–17.78%; *p* < 0.001). Bank voles (*Myodes glareolus*) and yellow-necked mice (*Apodemus flavicollis*) were the main reservoirs; synanthropic rodents trapped within the city were found to be infected for the first time. No significant organotropism was detected, but positive correlations between infection in the heart, liver, and kidney suggested hematogenous dissemination. The overall human seroprevalence of *B. miyamotoi* was 1.71% (95% CI: 1.34–2.18%) and was significantly higher in Leningrad Oblast (2.19%; 95% CI: 1.66–2.89%) than in Saint Petersburg (1.03%; 95% CI: 0.64–1.67%; *p* = 0.010). In contrast, the seroprevalence of *B. burgdorferi* s. l. did not differ between the two regions (approximately 5.1%). **Conclusions**: This first comprehensive, multi-level investigation in Saint Petersburg and Leningrad Oblast reveals a stable epidemiological gradient: natural foci in Leningrad Oblast sustain higher *B. miyamotoi* circulation in ticks and rodents, which translates into a two-fold higher exposure of the rural population. The findings highlight the need to include *B. miyamotoi* in regional tick-borne infection surveillance programs and to adopt differentiated risk assessment strategies for urban and rural settings.

## 1. Introduction

Tick-borne infections remain a significant public health challenge in the temperate zone of the Northern Hemisphere [1,2,3]. Alongside classical Lyme borreliosis (or Lyme disease) caused by bacteria of the *Borrelia burgdorferi* sensu lato complex, recent decades have witnessed growing interest in the etiological agents of tick-borne relapsing fever (TBRF) transmitted by hard ticks [4,5].

Among *Borrelia* species, *Borrelia miyamotoi* has attracted considerable scientific attention. In 2003 in Russia, the first ever confirmation of its role in human disease was obtained: DNA of this pathogen was detected in the blood of patients presenting with a febrile illness without erythema migrans [6,7]. Subsequently, these findings were corroborated by studies conducted in other countries, leading to the recognition of *B. miyamotoi* as a novel zoonotic pathogen [8]. The clinical presentation of *B. miyamotoi* infection is nonspecific, characterized by acute febrile illness, headache, chills, and myalgia, often accompanied by laboratory findings of thrombocytopenia and elevated hepatic transaminases [9,10]. In some cases, the disease follows a relapsing fever course with recurrent febrile episodes. This symptom complex creates substantial difficulties for differential diagnosis with other natural focal febrile illnesses, including hemorrhagic fever with renal syndrome, tick-borne encephalitis (TBE), anaplasmosis, and ehrlichiosis [6,11]. According to recent studies, *B. miyamotoi* is capable of causing severe pathological conditions. In particular, the development of meningoencephalitis has been documented in immunocompromised patients, and the etiological role of this pathogen in mixed infections with other tick-borne agents has been established [12,13,14,15].

Unlike the TBRF agents, which are transmitted by soft ticks (*Ornithodoros* spp.), *B. miyamotoi* is maintained in ixodid ticks of the genus *Ixodes*. Saint Petersburg and Leningrad Oblast, a region with widespread distribution of *I. ricinus* and *I. persulcatus*, is characterized by a high abundance of tick vectors and the sustained activity of natural foci of tick-borne infections. A distinctive feature of the epidemiological situation in this area is the overlapping distribution ranges of these two tick species, which creates conditions for the formation of mixed foci and a high risk of co-infection [16,17,18,19,20].

Climate change is increasingly recognized as a major driver of tick-borne disease emergence. In North America, modelling studies project a substantial increase in Lyme disease incidence attributable to climate change, with estimates suggesting up to a 20% rise over the next one to two decades [21]. Comparable projections for *Borrelia miyamotoi* are currently limited, but given its ecological similarity to *B. burgdorferi* s.l., a similar climate-driven expansion of its range and transmission season is plausible. In Russia, studies on climate change and tick-borne diseases have primarily focused on tick-borne encephalitis and Lyme borreliosis [22], but data on *B. miyamotoi* remain scarce. The Northwestern Federal District, with its overlapping ranges of *I. ricinus* and *I. persulcatus*, may be particularly vulnerable to climate-induced changes in tick distribution and pathogen circulation.

Transovarial transmission is considered the primary mechanism maintaining *B. miyamotoi* circulation in tick populations [23,24]. However, small mammals, particularly rodents, play a crucial role as reservoir hosts, supporting pathogen spread in natural foci [25,26,27]. Russia, especially its northwestern and Siberian regions, is recognized as endemic for *B. miyamotoi*. Serological and molecular genetic studies confirm the circulation of this pathogen in natural foci and the occurrence of human infections [27,28,29,30].

A major gap in the epidemiological assessment of natural foci is the lack of integrated studies covering all key components of the epidemic process (vectors, reservoir hosts, human population) simultaneously in the urban setting of Saint Petersburg and the diverse forested landscapes of Leningrad Oblast. Such an approach is essential for understanding the spatial distribution patterns of the pathogen, assessing the actual level of human exposure, and establishing a scientific basis for high-quality epidemiological surveillance. To date, no integrated studies combining data on infection rates in ticks, small mammals, and human seroprevalence have been conducted specifically for this part of northwestern Russia.

The aim of the study was to perform a comprehensive assessment of *B. miyamotoi* circulation in natural foci of Saint Petersburg and Leningrad Oblast. This was achieved through a coupled analysis of infection prevalence in tick vectors and small mammal reservoirs, alongside the determination of seroprevalence for this pathogen among the population of key constituent entities of the region.

## 2. Materials and Methods

### 2.1. Study Design and Period

This study was conducted in Saint Petersburg and Leningrad Oblast, Russia. Field samples of ixodid ticks and small mammals were collected during the active seasons of 2022–2024. Serum samples from healthy volunteers were obtained in 2023.

### 2.2. Serum Sampling from the Population and Ethical Considerations

Serum samples were collected in September 2023 within the framework of the Federal Service for the Oversight of Consumer Protection and Welfare (Rospotrebnadzor) program “Assessment of herd immunity to vaccine-preventable and other relevant infections in the population of Saint Petersburg and Leningrad Oblast”, from randomly selected volunteers. Volunteers were informed about the study purpose and signed voluntary informed consent forms. The study protocol was approved by the Local Ethics Committee of the Saint Petersburg Pasteur Institute (Protocol No. 86, 17 August 2023). Venous blood (3 mL) was drawn from the cubital vein into serum separation tubes. Serum was separated by centrifugation at 1500× *g* for 10 min at room temperature, aliquoted, and stored at −20 °C until analysis.

In addition to blood sampling, all participating volunteers completed a standardized epidemiological questionnaire. The questionnaire collected information on age, sex, place of permanent residence, history of tick bites within the last 5 years (number of bites, if any), and vaccination status against tick-borne encephalitis (TBE). For TBE vaccination, individuals with a documented history of at least one completed vaccination course were considered “vaccinated”. The groups “tick bite in the last 5 years” and “TBE vaccinated” were not mutually exclusive. A total of 3743 serum samples were obtained from apparently healthy individuals permanently residing in Saint Petersburg (*n* = 1553) and Leningrad Oblast (*n* = 2190).

### 2.3. Collection Sites for Entomological and Zoological Material

**Figure 1:**
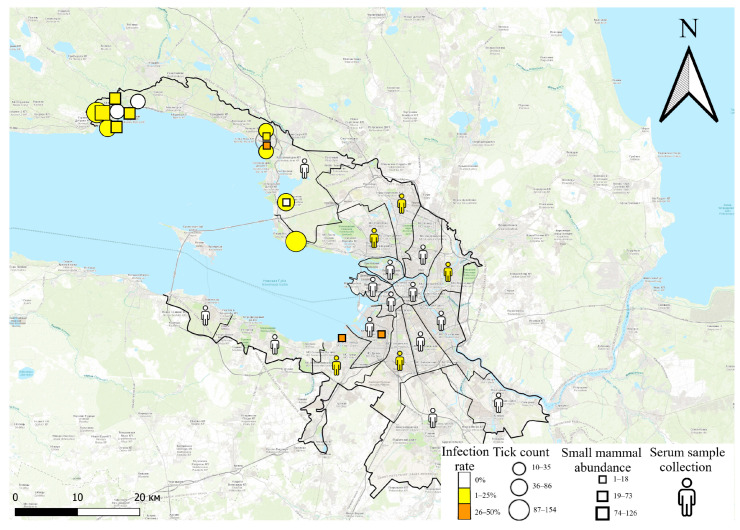
Map of Saint Petersburg depicting the spatial distribution of small mammal trapping sites and tick collection sites. Square size represents small mammal abundance; circle size corresponds to total number of ticks collected. Fill color indicates *B. miyamotoi* infection rates in collected specimens.

**Figure 2:**
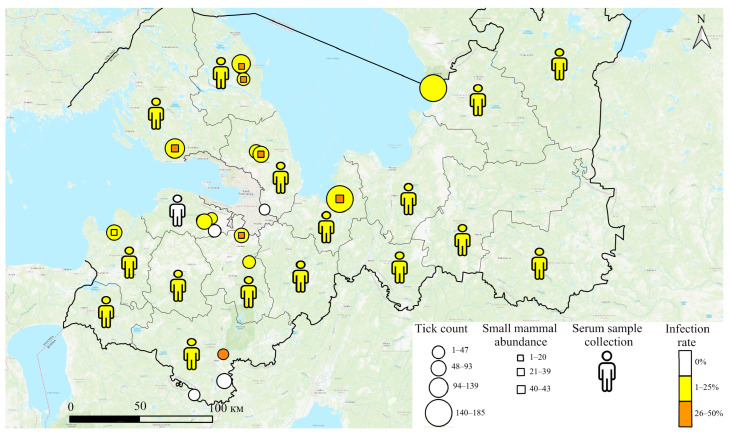
Map of Leningrad Oblast showing sampling sites for small mammals and ticks. Square size represents small mammal abundance; circle size represents total number of ticks collected. Fill color indicates *B. miyamotoi* infection rates in collected specimens.

Tick collection and small mammal trapping were focused on recreational and natural areas in the city of Saint Petersburg and Leningrad Oblast. The geographic distribution of sampling sites, along with small mammal abundance and tick collection numbers, is shown in Figure 1 (Saint Petersburg) and Figure 2 (Leningrad Oblast). The fill color of the symbols in both figures indicates *B. miyamotoi* infection rates in ticks and small mammals.

### 2.4. Tick Collection and Processing

Ticks were collected during clear weather, primarily in meadow and forest areas, using a 60 × 100 cm flannel flag. A 1 km transect length was used as the unit of recording. Collected ticks were placed alive into individual 2.0 mL screw-cap Eppendorf tubes and transported to the laboratory at ambient temperature. Tick species and sex were identified based on morphological characteristics following established guidelines [31]. Each tick was processed and examined individually. A total of 1518 unfed adult ticks were collected. In Saint Petersburg, 582 ticks were collected, including 345 *I. ricinus* and 237 *I. persulcatus*. In Leningrad Oblast, 924 ticks were collected, including 506 *I. ricinus* and 418 *I. persulcatus*.

### 2.5. Trapping, Identification, and Organ Sampling of Small Mammals

Wild small mammals were trapped using Gero live traps. Pieces of bread crust coated with vegetable oil were used as bait. After capture, animals were identified to species based on morphological characteristics following established guidelines [32]. All trapped species are considered non-threatened and are not protected under regional or international conservation regulations. Carcasses were placed in individual cotton bags with accompanying labels and transported deceased to the laboratory on the day of capture. During transport, samples were kept at −20 °C and subsequently stored at −80 °C until necropsy and collection of internal organ samples (heart, liver, spleen, kidneys, and brain).

A total of 516 small mammals were trapped. In Saint Petersburg, 374 specimens were captured, including wild species: *Myodes glareolus*—262, *Apodemus flavicollis*—93, *Sorex araneus*—6, *Apodemus uralensis*—4, and *Microtus arvalis*—3. Synanthropic species were trapped in the Kirovsky District (*Rattus norvegicus*—1) and in the Krasnoselsky District (*Mus musculus*—5). Additionally, single specimens of small mammals were trapped in the Kirovsky (59.871111, 30.273309) and Krasnoselsky (59.867589, 30.154204) districts of the city. In Leningrad Oblast, 142 wild small mammals were captured, including *Myodes glareolus*—66, *Apodemus flavicollis*—45, *Sorex araneus*—7, *Apodemus uralensis*—11, *Apodemus agrarius*—5, *Micromys minutus*—5, *Microtus arvalis*—2, and one specimen of *Talpa europaea*.

### 2.6. Sample Preparation and Nucleic Acid Extraction

All tick and organ samples were examined individually. Specimens were placed into individual tubes containing 400 μL of sterile phosphate-buffered saline (PBS). Two sterile 4.5 mm steel beads were added to each tube. Samples were homogenized using a FastPrep-24 mechanical homogenizer (MP Biomedicals, Santa Ana, CA, USA) at 4 m/s for 1 min. Following centrifugation of homogenates (13,600× *g* for 1 min), 100 μL of supernatant was collected for nucleic acid (NA) extraction. NA extraction was performed using the RIBO-prep reagent kit (FBIS CRIE Rospotrebnadzor, Moscow, Russia) according to the manufacturer’s instructions.

### 2.7. Borrelia miyamotoi Real-Time PCR

Detection of *B. miyamotoi* DNA was performed by real-time PCR using a CFX96 C1000 Touch™ thermal cycler (Bio-Rad, Hercules, CA, USA) and the commercial reagent kit “AmpliSens^®^ *Borrelia miyamotoi*-FL” (FBIS CRIE Rospotrebnadzor, Moscow, Russia).

### 2.8. Serological Analysis

All serum samples were tested for the presence of specific antibodies (IgG and IgM) against *B. miyamotoi* and *B. burgdorferi* s.l. using a protein microarray, following previously published methods [33,34]. Serum samples were considered positive for *B. miyamotoi* IgM and IgG in the case of seroreactivity to GlpQ and any of the Vmp antigens: Vsp1, Vlp5 (Vlp-γ), Vlp15/16 (Vlp-δ), Vlp18 (Vlp-α) or flagellin (p41), and p39 or BBK32. Samples seroreactive only to GlpQ were considered equivocal [16]. In order to simplify subsequent calculations and ensure the accuracy of the results, any questionable results were treated as negative.

Samples positive for *B. burgdorferi* s.l. IgM were defined by seroreactivity simultaneously to OspC and to at least one of the following antigens (p100, VlsE, p41, p39, p58, BBK32 p17), or to at least three of the following antigens (p100, VlsE, p41, p39, p58, BBK32 p17). Seropositivity for *B. burgdorferi* s.l. IgG was defined as seroreactivity to VlsE and to at least one of the following antigens (p41, p100, p58, p39, BBK32, OspC, p17) or to at least three of the following antigens (p100, p41, p39, p58, BBK32, OspC, p17) [34].

### 2.9. Statistical Analysis

No formal sample size calculation was performed a priori. The sample size for human seroprevalence was determined by the availability of serum samples collected within the Rospotrebnadzor herd immunity program. For tick and small mammal collections, sample sizes were determined by the feasibility of field sampling during the study period. These sample sizes are comparable to or exceed those reported in similar seroprevalence studies on tick-borne pathogens.

Infection rates with 95% confidence intervals (CIs) were calculated using the Wilson score method. A two-sided Z-test was used to compare proportions between two groups. Associations between categorical variables (species, sex, district) and infection status were assessed using Pearson’s chi-square (χ^2^) test. Correlations between organ infection status in rodents were evaluated by constructing a correlation matrix using the phi coefficient. All calculations were performed in the Python 3.12.0 programming environment using the SciPy and Pandas libraries. The level of statistical significance was set at *p* < 0.05.

To assess potential risk factors and demographic patterns, subgroup analyses were conducted. These included comparisons of *B. miyamotoi* infection rates between tick species (*I. ricinus* vs. *I. persulcatus*), between collection sites (Leningrad Oblast vs. Saint Petersburg), and among small mammal species. For human seroprevalence, subgroup analyses according to donor sex, age group, and region of residence (Leningrad Oblast vs. Saint Petersburg) were performed. Age groups were stratified by decade, with criteria defined post hoc based on sample distribution to ensure sufficient group sizes for statistical comparison.

## 3. Results

### 3.1. Infection Rate in Ixodid Ticks

**Figure 3:**
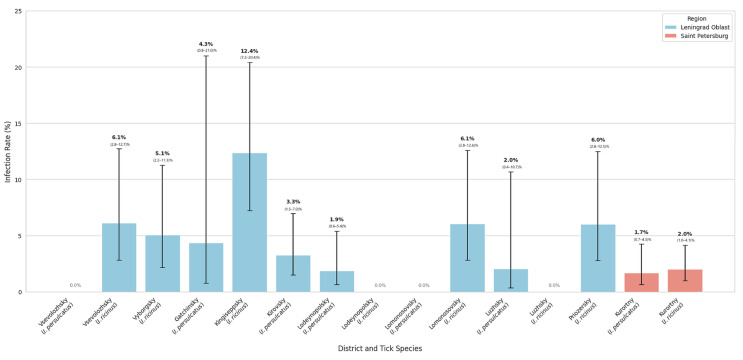
*B. miyamotoi* infection rates in ixodid ticks across administrative districts of Leningrad Oblast and Saint Petersburg.

Based on individual examination of ixodid ticks collected in northwestern Russia, the overall prevalence of *B. miyamotoi* was 3.78% (57/1506). For *I. persulcatus*, the infection rate was 2.29% (95% CI: 1.39–3.74%). For *I. ricinus*, the rate was 4.94% (95% CI: 3.67–6.60%). *I. ricinus* showed a significantly higher infection rate than *I. persulcatus* (*p* = 0.011). Ticks collected in Leningrad Oblast had an infection rate of 4.98% (95% CI: 3.75–6.58%), which is markedly higher than the rate in Saint Petersburg: 1.89% (95% CI: 1.06–3.35%). The chi-square test revealed a significant difference in infection rates between the two regions (*p* = 0.004). Detailed data are shown in Figure 3.

The bar chart illustrates the prevalence of *B. miyamotoi* in dominant tick species (*I. ricinus* and *I. persulcatus*) collected from various sampling points. Infection rates are expressed as percentages with accompanying 95% Wilson confidence intervals (CIs). Numerical values above the bars indicate the point estimate of the infection rate and the specific CI range.

Key findings include a significant geographical heterogeneity in pathogen prevalence. The highest infection rate was observed in *I. ricinus* from the Kingiseppsky district of Leningrad Oblast (12.4%, 95% CI: 7.2–20.4%). Moderate prevalence (approximately 6.0%) was noted in the Vsevolozhsky, Lomonosovsky, and Priozersky districts. In Saint Petersburg (Kurortny district), infection rates remained relatively stable at approximately 1.7–2.0% for both *I. persulcatus* and *I. ricinus*. Error bars are omitted for locations with zero prevalence due to the absence of positive samples.

### 3.2. Infection Rate of Small Mammals

**Figure 4:**
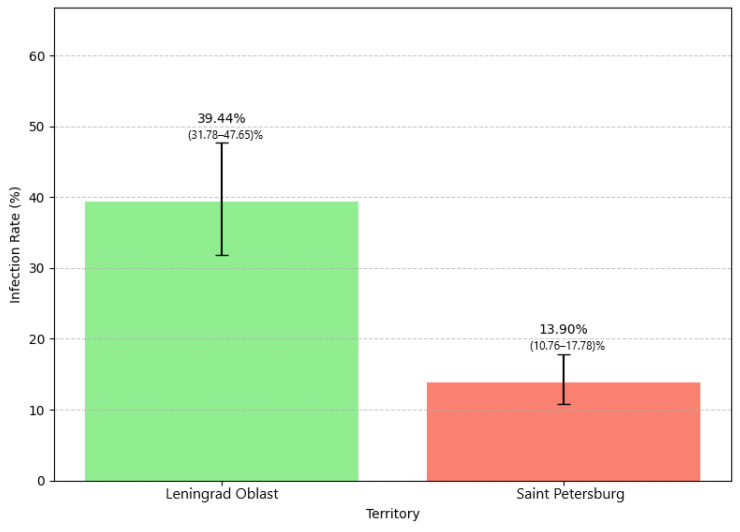
*B. miyamotoi* infection rates in small mammals by capture territory.

*B. miyamotoi* DNA was detected in 20.93% (108/516) of small mammals (95% CI: 17.64–24.65%). The infection rate of small mammals by capture region is shown in Figure 4. Small mammals trapped in Leningrad Oblast had a significantly higher infection rate (39.44%; 95% CI: 31.78–47.65%) compared to those from Saint Petersburg (13.90%; 95% CI: 10.76–17.78%; χ^2^ = 39.02, *p* < 0.001).

A statistically significant variation in infection rate was observed among species (χ^2^ = 46.62, *p* < 0.001). High infection levels were noted in *M. arvalis* (5/5; 100%), *M. minutus* (5/5; 100%), *R. norvegicus* (1/1; 100%), and *T. europaea* (1/1; 100%). However, confidence intervals for these species were very wide due to the small sample sizes. *M. musculus* (3/5; 60.00%; 95% CI: 23.07–88.24), *A. agrarius* (2/5; 40%; 95% CI: 13.68–69.43), *A. uralensis* (5/15; 33.33%; 95% CI: 15.18–58.29), *M. glareolus* (65/328; 19.82%; 95% CI: 15.86–24.47), and *A. flavicollis* (19/138; 13.77%; 95% CI: 8.99–20.50) showed varying infection levels.

### 3.3. Organ Distribution in Rodents

**Figure 5:**
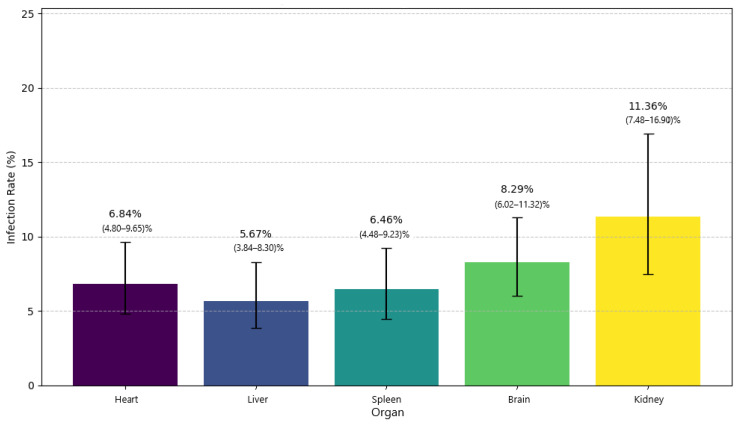
*B. miyamotoi* infection rates in internal organs of small mammals.

To assess the organotropism of *B. miyamotoi* in small mammals, specimens with at least four organs examined simultaneously were selected from the overall sample. The results of this analysis are shown in Figure 5.

The highest infection rate was detected in the kidneys: 11.36% (95% CI: 7.48–16.90%). Other organs were infected to a lesser extent: 8.29% in the brain (95% CI: 6.02–11.32%); 6.84% in the heart (95% CI: 4.80–9.65%); 6.46% in the spleen (95% CI: 4.48–9.23%); and 5.67% in the liver (95% CI: 3.84–8.30%). The chi-square test did not reveal statistically significant differences in infection rates between organs (χ^2^ = 7.17, *p* = 0.127).

**Figure 6:**
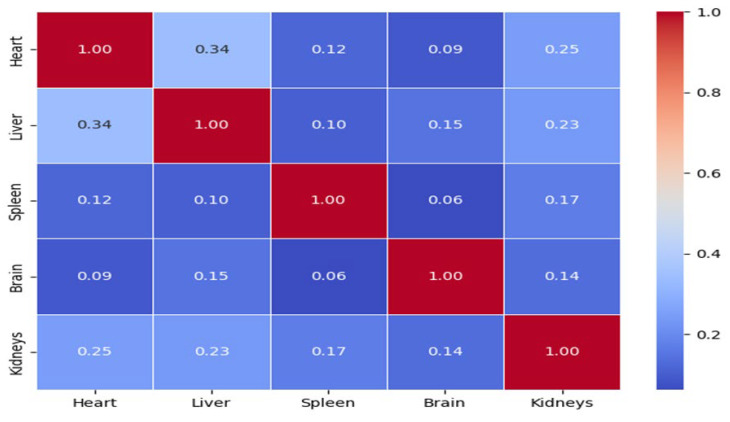
Pearson correlation matrix for *B. miyamotoi* infection in internal organs of small mammals.

To assess the relationship between infection status in different organs, a correlation analysis was performed (Figure 6).

The correlation matrix revealed positive correlations between infections in the heart, liver, and kidneys (with correlation coefficients ranging from 0.23 to 0.34). Infections in the brain and spleen showed weak or no correlation with other organs.

### 3.4. B. miyamotoi Seroprevalence in the Healthy Population of Saint Petersburg and Leningrad Oblast

**Figure 7:**
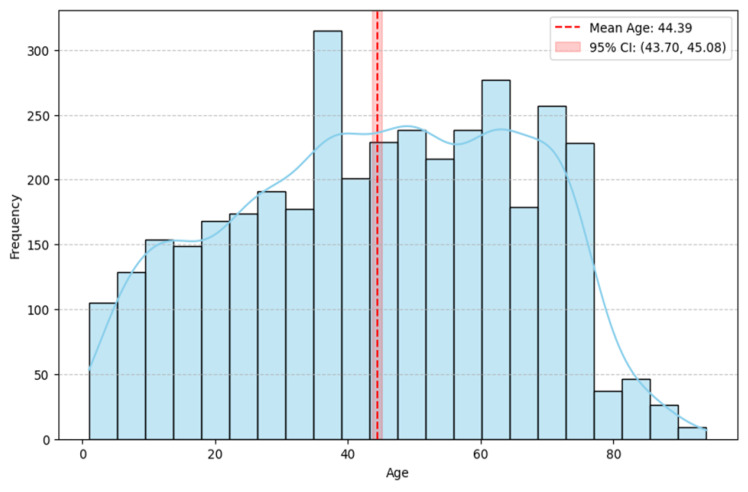
Age distribution of volunteers.

To characterize the study population, we analyzed the age composition of the volunteers (Figure 7). The histogram illustrates a broad age range in the sample, from 1 to 94 years. The distribution is polymodal, with distinct peaks in the middle and older adult age groups (40–60 years), indicating the highest representation of these age categories in the sample. The frequency gradually decreases towards the extremes of the age spectrum.

In contrast to age, the sex distribution was skewed, with a notable predominance of female participants. Female volunteers (*n* = 2748) had a mean age of 46.1 years (median 47.0 years). Male volunteers (*n* = 995) had a mean age of 39.6 years (median 40.0 years). The sample included individuals aged 1 to 94 years for both sexes, with female volunteers on average approximately 6.5 years older than male volunteers.

To assess population *B. miyamotoi* seroprevalence, serum samples were obtained from apparently healthy individuals permanently residing in Saint Petersburg (*n* = 1553) and the Leningrad Oblast (*n* = 2190), with the latter representing predominantly rural and suburban populations. Overall *B. miyamotoi* seroprevalence was 1.71% (95% CI: 1.34–2.18%). We then analyzed this indicator by volunteer region of residence. In Leningrad Oblast, seroprevalence was 2.19% (95% CI: 1.66–2.89%). In Saint Petersburg, this indicator was significantly (χ^2^ = 6.62, *p* = 0.010) lower: 1.03% (95% CI: 0.64–1.67%).

In St. Petersburg, individuals with a history of tick bites in the past five years featured significantly higher *B. burgdorferi* s.l. IgG seroprevalence (6.8%, *p* < 0.05) compared to those without reported bites. In Leningrad Oblast, the overall seroprevalence of *B. miyamotoi* (IgM or IgG) was significantly higher (2.1%, *p* < 0.001) than in St. Petersburg, with a notable contribution from IgM-positive cases (0.7%, *p* < 0.01). No significant associations were observed between TBE vaccination status and seropositivity to either *Borrelia* species, suggesting the absence of cross-reactivity or vaccine-induced immunological interference (Table 1A,B).

In samples collected in St. Petersburg and Leningrad Oblast, the prevalence of IgM to *B. burgdorferi* s.l. was 1.9% (95% CI: 1.3–2.8) and 2.1% (95% CI: 1.5–2.8). The prevalence of IgG to *B. burgdorferi* s.l. was 3.2% (95% CI: 2.3–4.2) and 3.0% (95% CI: 2.3–3.8), respectively. Higher IgG seroprevalence (6.8%, 95% CI: 3.9–11.0), compared to all samples (*p* = 0.0065), was found in samples of conditionally healthy donors who had previously experienced tick bites living in St. Petersburg.

In St. Petersburg and Leningrad Oblast, respectively: the prevalence of IgM to *B. miyamotoi* was 0.09% (95% CI: 0.03–0.4) and 0.7% (95% CI: 0.4–1.2); and the prevalence of IgG to *B. miyamotoi* was 1.0% (95% CI: 0.5–1.6) and 1.4% (95% CI: 1.0–2.0). Interestingly, IgM and IgM/IgG seroprevalence was significantly lower in samples collected in St. Petersburg (** *p* = 0.0029 and *** *p* = 0.012, respectively).

IgG seroprevalence for *B. burgdorferi* s.l. was significantly higher in men than in women in Leningrad Oblast (*p* = 0.03). A higher IgG seroprevalence was also observed in the oldest age group (70–94 years) in St. Petersburg, although this difference did not reach statistical significance (*p* = 0.08). Sex-stratified (Table S1), age-stratified (Table S2), and district-level seroprevalence data (Table S3) are provided in the Supplementary Materials.

Seroprevalence (IgM and/or IgG) for *B. burgdorferi* s.l. was significantly higher among residents of the Boksitogorsky and Podporozhsky districts of Leningrad Oblast compared to the regional average (** *p* = 0.05). The seroprevalence (IgM and/or IgG) for *B. miyamotoi* was significantly higher in the Kirovsky district of Leningrad Oblast (*** *p* = 0.03) and the Krasnogvardeysky district of St. Petersburg (**** *p* = 0.04) compared to their respective regional averages.

**Figure 8:**
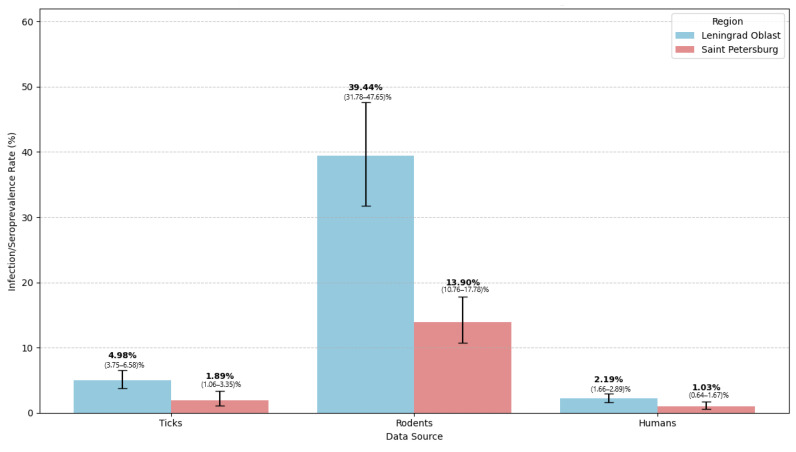
Comparative analysis of *B. miyamotoi* infection rates in ticks, small mammals, and human seroprevalence by region.

For a comprehensive assessment of region endemicity and investigation of the pathogen’s ecological cycle, a comparative analysis was performed combining data from all studied components: infection rates in tick vectors, infection prevalence in reservoir hosts, and seroprevalence in humans. The results are presented as a grouped bar chart in Figure 8.

Thus, a clear and statistically significant gradient of *B. miyamotoi* infection/infestation levels was identified in both regions. Rodents featured the highest rates: 39.44% (95% CI: 31.78–47.65%) in Leningrad Oblast and 13.90% (95% CI: 10.76–17.78%) in Saint Petersburg. Ticks occupied an intermediate position: 4.98% (95% CI: 3.75–6.58%) in Leningrad Oblast and 1.89% (95% CI: 1.06–3.35%) in Saint Petersburg. Humans had the lowest seroprevalence rates: 2.19% (95% CI: 1.66–2.89%) in Leningrad Oblast and 1.03% (95% CI: 0.64–1.67%) in Saint Petersburg.

## 4. Discussion

This study provides a comprehensive, multi-level assessment of *B. miyamotoi* circulation in northwestern Russia, integrating data from tick vectors, small mammal reservoirs, and human populations. Our findings reveal a consistent epidemiological gradient: higher pathogen infection rates in ticks and small mammals in the Leningrad Oblast compared to Saint Petersburg, which translates into a significantly higher human seroprevalence in the rural population. In contrast, *B. burgdorferi* s.l. seroprevalence was similar between the two regions, suggesting distinct ecological drivers.

The overall infection rate (3.78%) is consistent with reports from other countries, where *B. miyamotoi* infection in ticks under natural conditions is typically under 5% [23]. However, the rate observed in our study was higher than that reported, for example, in neighboring Finland, where tick infection prevalence was 0.7% [35,36]. Despite this, the level of *B. miyamotoi* infection in ixodid ticks in Saint Petersburg and Leningrad Oblast is considerably lower than previously published data on *B. burgdorferi* s.l. infection, which is 23% [17,18]. District-level analysis revealed significant variability, with high infection rates in some districts (e.g., Kingiseppsky, 15.91%). Infection rates also differed significantly between *I. persulcatus* and *I. ricinus* (χ^2^ = 6.40, *p* = 0.011), confirming that *I. ricinus* is the primary vector, consistent with European studies [37,38]. Further studies are needed to investigate these differences.

The overall infection rate in small mammals was 20.93% (95% CI: 17.64–24.65), significantly higher than reported elsewhere [38,39,40]. Bank voles (*M. glareolus*) and yellow-necked mice (*A. flavicollis*) were the main reservoirs, consistent with European data [39,40,41,42]. Notably, this study provides the first data on *B. miyamotoi* infection in synanthropic rodents (*M. musculus*, *R. norvegicus*) trapped within Saint Petersburg.

No significant tissue tropism was detected for specific internal organs of small mammals (χ^2^ = 7.17, *p* = 0.127). The highest infection rate was observed in the kidneys (11.36%), although this difference was not statistically significant. One possible explanation is that the kidneys may serve as a site of persistent infection or immune privilege for relapsing fever borreliae. Alternatively, the higher detection rate in kidney tissue could reflect tropism for the vascular endothelium, which is abundant in renal microvasculature. Further studies using histopathological or culture-based methods are needed to clarify this observation [43]. Correlation analysis revealed moderate positive correlations between the heart, liver, and kidneys (r ≈ 0.25–0.34). This suggests hematogenous dissemination, a feature that distinguishes tick-borne relapsing fever group pathogens from *B. burgdorferi* s.l., which typically remains localized [44].

Northwestern Russia is endemic for many tick-borne pathogens [19,20,45]. The overall seroprevalence rate was 1.71% (95% CI: 1.34–2.18), which is comparable to the rate for apparently healthy individuals obtained in a similar study in Denmark: 1.5% (95% CI: 0.8–2.8) [46]. The IgM/IgG seroprevalence of *B. miyamotoi* in healthy population controls established by the same method was 2.5% (95% CI: 1.5–4.1) in the Netherlands and 1.0% (95% CI: 0.1–6.9) in Sweden [47]. This similarity suggests broadly consistent epidemiological patterns of *B. miyamotoi* circulation in the temperate climatic zone of Europe. Compared to the seroprevalence of other endemic tick-borne infections characteristic of northwestern Russia, this rate is lower than that for *B. burgdorferi* s.l. (3.7%) and *E. chaffeensis*/*E. muris* (3.2%) [19]. This divergence could be partly attributed to differences in the competent reservoir host structure. Urban forest parks, where a substantial portion of the population is exposed to ticks, are often dominated by a limited number of small mammal and avian species that serve as highly efficient reservoirs for *B. burgdorferi* s.l. but remain suboptimal for *B. miyamotoi*. Conversely, the diverse wildlife communities in less disturbed forest landscapes provide the stable multi-host system required for robust *B. miyamotoi* circulation, thereby limiting high-density pathogen amplification near heavily populated urban centers.

In our study, *B. burgdorferi* s.l. seroprevalence was 5.16% (95% CI: 4.31–6.17), approximately one percentage point higher than previously reported. This difference may reflect the higher analytical sensitivity of the microarray, which detects low-avidity antibodies, and our inclusion of both IgM and IgG. The comparable study considered IgG only [19]. A true increase due to tick range expansion cannot be ruled out, but results should be interpreted with caution due to different assay methods.

Analysis of demographic factors revealed that, despite a female skew in the sample, sex-specific analysis showed a trend towards higher IgG seroprevalence for both pathogens in males, particularly in Leningrad Oblast (*p* = 0.03 for *B. burgdorferi* s.l. IgG). This is consistent with higher occupational or recreational tick exposure in men [48]. Age-specific seroprevalence showed expected accumulation of exposure over time, with the highest IgG levels in the age group of 70–94 years for both pathogens. Detectable IgM responses across a wide age range, including children, confirm active pathogen circulation in natural foci accessible to all age groups.

A notable finding was the marked urban–rural heterogeneity for *B. miyamotoi*: seroprevalence in Leningrad Oblast (2.19%, 95% CI: 1.66–2.89) was more than twice that in Saint Petersburg (1.03%, 95% CI: 0.64–1.67, *p* = 0.010). In contrast, *B. burgdorferi* s.l. seroprevalence was virtually identical between the two regions (≈5.1%). This contrasting pattern likely reflects differences in vector ecology and behavioral risk factors. *B. burgdorferi* s.l., circulating predominantly in *I. persulcatus*, is widely distributed across the region, including urban forest parks [17,18,45], explaining uniformly high seroprevalence. In contrast, *B. miyamotoi*, transmitted transovarially and associated with less disturbed natural foci, is more strongly linked to rural biotopes [23]. The higher seroprevalence in Leningrad Oblast may reflect more frequent or prolonged exposure during occupational or recreational activities. Alternative explanations, such as differences in immune response during asymptomatic infection, require further study.

Together, these data provide robust evidence of a consistent epidemiological gradient for *B. miyamotoi* across the studied regions. The consistent direction of regional differences across all three levels of the epidemic process (rodent, tick, human) supports the robustness of the findings and argues against sampling bias.

## 5. Conclusions

This study provides the first serological assessment of population immunity to *B. miyamotoi* in northwestern Russia using protein microarray technology. The findings reveal a clear association between pathogen circulation in natural foci and human exposure to the pathogen. The observed urban–rural gradient highlights the need for region-specific risk assessment. Average seroprevalence estimates for a metropolitan area and its surrounding regions may obscure substantial local differences that should be considered in prevention planning. These findings support the inclusion of *B. miyamotoi* in regional tick-borne pathogen surveillance programs and provide a baseline for future monitoring of this emerging pathogen in northwestern Russia.

## Figures and Tables

**Table 1:** (A) *B. miyamotoi* seroprevalence by TBE vaccination status and history of a tick bite in the past 5 years. (B) *B. burgdorferi* s.l. seroprevalence by TBE vaccination status and history of a tick bite in the past 5 years.

**(A)**					
**Region**	**Individuals, *N***	**Anti-*B. miyamotoi* Antibodies**			
		**IgM**	**IgG**	**IgM or IgG**	**IgM and IgG**
		**Number of Positive Samples, *n*, % (95% CI)**			
St. Petersburg	Total, 1553	1 0.0 (0.0–0.4)	15 1.0 (0.5–1.6)	16 1.0 (0.6–1.7)	0 0.0 (0.0–0.2)
	Tick-bite last 5 years, 236	0 0.0 (0.0–1.5)	3 1.3 (0.3–3.7)	3 1.3 (0.3–3.7)	0 0.0 (0.0–1.5)
	TBE vaccination, 269	0 0.0 (0.0–1.4)	1 0.4 (0.0–2.1)	0 0.0 (0.0–1.4)	0 0.0 (0.0–1.4)
Leningrad Oblast	Total, 2190	16 ** 0.7 (0.4–1.2)	31 1.4 (1.0–2.0)	46 *** 2.1 (1.5–2.8)	1 0.0 (0.0–0.3)
	Tick-bite last 5 years, 256	1 0.4 (0.0–2.2)	6 2.3 (0.9–5.1)	7 2.7 (1.1–5.6)	0 0.0 (0.0–1.4)
	TBE vaccination, 474	6 1.3 (0.5–2.8)	4 0.8 (0.2–2.2)	10 2.1 (1.0–3.9)	0 0.0 (0.0–0.8)
**(B)**					
**Region**	**Individuals, *N***	**Anti-*B. burgdorferi*** **s.l Antibodies**			
		**IgM**	**IgG**	**IgM or IgG**	**IgM and IgG**
		**Number of Positive Samples, *n*, % (95% CI)**			
St. Petersburg	Total, 1553	30 1.9 (1.3–2.8)	49 3.2 (2.3–4.2)	75 4.8 (3.8–6.0)	4 0.3 (0.0–0.6)
	Tick-bite last 5 years, 236	4 1.7 (0.5–4.3)	16 * 6.8 (3.9–11.0)	18 7.6 (4.5–12.1)	2 0.9 (0.1–3.1)
	TBE vaccination, 269	7 2.6 (1.0–5.4)	12 4.5 (2.3–7.8)	18 6.7 (4.0–10.6)	1 0.4 (0.0–2.1)
Leningrad Oblast	Total, 2190	46 2.1 (1.5–2.8)	66 3.0 (2.3–3.8)	110 5.0 (4.1–6.0)	2 0.1 (0.1–0.3)
	Tick-bite last 5 years, 256	7 2.7 (1.1–5.6)	9 3.5 (1.6–6.7)	15 5.9 (3.3–9.7)	1 0.4 (0.0–2.2)
	TBE vaccination, 474	16 3.4 (1.9–5.5)	9 1.9 (0.9–3.6)	24 5.1 (3.2–7.5)	1 0.2 (0.0–1.1)

Note: Data are presented as *n* (%) with 95% confidence intervals (Clopper–Pearson method). Seropositivity criteria are described in Section 2.8. Definitions of “Tick bite in the last 5 years” and “TBE vaccinated” groups are provided in Section 2.2. These groups are not mutually exclusive. Asterisks indicate statistically significant differences compared to the rest of the regional population (* *p* < 0.05, ** *p* < 0.01, *** *p* < 0.001).

## Data Availability

The original contributions presented in this study are included in the article. Further inquiries can be directed to the corresponding author.

## Supplementary Materials

The following supporting information can be downloaded at: https://www.mdpi.com/article/10.3390/tropicalmed11060166/s1, Table S1: Seroprevalence of *Borrelia burgdorferi* sensu lato and *Borrelia miyamotoi* among the population of St. Petersburg and Leningrad Oblast, stratified by region and sex; Table S2: Age–stratified seroprevalence of *Borrelia burgdorferi* s.l. and *Borrelia miyamotoi*; Table S3: Seroprevalence of *Borrelia* species in different administrative districts of St. Petersburg and Leningrad Oblast.

## Abbreviations

The following abbreviations are used in this manuscript:

PCR	Polymerase chain reaction
DNA	Deoxyribonucleic acid
TBRF	Tick-borne relapsing fever
TBE	Tick-borne encephalitis
CI	Confidence intervals
NA	Nucleic acid
