# Supplementary material for: Borrelia miyamotoi in Saint Petersburg and Leningrad Oblast, Russia: A Multi-Level Assessment of Ticks, Rodents, and Human Exposure
Source: Trop Med Infect Dis. 2026 Jun 18;11(6):166. doi: 10.3390/tropicalmed11060166 (PMC13307801; doi:10.3390/tropicalmed11060166)
Supplement: Supplementary file 1 [file tropicalmed-11-00166-s001.zip › tropicalmed-4359725-supplementary.pdf]

Table S1. Seroprevalence of *Borrelia burgdorferi* sensu lato and *Borrelia miyamotoi* among the population of St. Petersburg and Leningrad Oblast, stratified by region and sex.

Region	Sex	Number of patients, N	*B. burgdorferi* s.l.		*B. miyamotoi*	
			IgM, *n*, % (95% CI)	IgG, n, % (95% CI)	IgM, n, % (95% CI)	IgG, n, % (95% CI)
St. Petersburg	F	1130	21<br>1.9 (1.2–2.8)	32<br>2.8 (1.9–4.0)	1<br>0.1 (0.0–0.5)	9<br>0.8 (0.4–1.5)
	M	406	9<br>2.2 (1.0–4.2)	16<br>3.9 (2.3–6.4)	0<br>0.0 (0.0–0.9)	6<br>1.5 (0.5–3.2)
Leningrad Oblast	F	1610	37<br>2.3 (1.6–3.2)	41<br>2.5 (1.8–3.5)	13<br>0.8 (0.4–1.4)	21<br>1.3 (0.8–2.0)
	M	579	9<br>1.6 (0.7–3.0)	25*<br>4.3 (2.8–6.4)	3<br>0.5 (0.1–1.5)	10<br>1.7 (0.8–3.2)

Note: Data are presented as number of positive cases (n) and percentage (%) with 95% confidence intervals (CI) in brackets. Confidence intervals for proportions were calculated using the Pearson exact method. Seropositivity was defined as the presence of specific IgM and/or IgG antibodies against the respective pathogen, as determined by microarray. No statistically significant differences between sexes within each region were observed for either pathogen, except for *B. burgdorferi* s.l. IgG in Leningrad Oblast, where males showed a higher seroprevalence compared to females ($p < 0.05$, chi–square test).

Table S2. Age–stratified seroprevalence of *Borrelia burgdorferi* s.l. and *Borrelia miyamotoi*.

Region	age	Number of patients, N	*B. burgdorferi* s.l		*B. miyamotoi*	
			IgM, n, % (95% CI)	IgG, n, % (95% CI)	IgM, n, % (95% CI)	IgG, n, % (95% CI)
St. Petersburg	1–5	42	2<br>4.8 (0.5–17.2)	0<br>0.0 (0.0–8.8)	0<br>0.0 (0.0–8.8)	1<br>2.4 (0.0–13.3)
	6–11	100	3<br>3.0 (0.6–8.8)	3<br>3.0 (0.6–8.8)	0<br>0.0 (0.0–3.7)	0<br>0.0 (0.0–3.7)
	12–17	76	2<br>2.6 (0.3–9.5)	1<br>1.3 (0.0–7.3)	0<br>0.0 (0.0–4.8)	0<br>0.0 (0.0–4.8)
	18–29	211	5<br>2.4 (0.8–5.2)	6<br>2.8 (1.0–6.2)	1<br>0.5 (0.0–2.6)	0<br>0.0 (0.0–1.8)
	30–39	206	7<br>3.4 (1.4–7.0)	4<br>1.9 (0.5–5.0)	0<br>0.0 (0.0–1.8)	3<br>1.5 (0.3–4.3)
	40–49	209	4<br>1.9 (0.5–4.9)	6<br>2.9 (1.0–6.2)	0<br>0.0 (0.0–1.8)	2<br>1.0 (0.1–3.4)
	50–59	208	0<br>0.0 (0.0–1.8)	5<br>2.4 (0.8–5.6)	0<br>0.0 (0.0–1.8)	4<br>1.9 (0.5–4.9)
	60–69	234	3<br>1.3 (0.2–3.8)	7<br>3.0 (1.2–6.2)	0<br>0.0 (0.0–1.6)	1<br>0.4 (0.0–2.4)
	70–94	250	4<br>1.6 (0.4–4.1)	16*<br>6.4 (3.7–10.4)	0<br>0.0 (0.0–1.5)	4<br>1.6 (0.4–4.1)

Leningrad Oblast	1–5	63	1<br>1.6 (0.0–8.8)	4<br>6.3 (1.7–16.3)	1<br>1.6 (0.0–8.8)	0<br>0.0 (0.0–5.9)
	6–11	101	3<br>3.0 (0.6–8.7)	2<br>2.0 (0.2–7.2)	0<br>0.0 (0.0–3.7)	0<br>0.0 (0.0–3.7)
	12–17	155	3<br>1.9 (0.4–5.7)	6<br>3.9 (1.4–8.4)	0<br>0.0 (0.0–2.4)	2<br>1.3 (0.2–4.7)
	18–29	284	10<br>3.5 (1.7–6.5)	1<br>0.4 (0.0–2.0)	2<br>0.7 (0.0–2.5)	1<br>0.4 (0.0–2.0)
	30–39	318	8<br>2.5 (1.1–5.0)	7<br>2.2 (0.9–4.5)	2<br>0.6 (0.0–2.3)	6<br>1.9 (0.6–4.1)
	40–49	321	5<br>1.6 (0.5–3.6)	4<br>1.2 (0.3–3.2)	3<br>0.9 (0.2–2.7)	5<br>1.6 (0.5–3.6)
	50–59	320	9<br>2.8 (1.3–5.3)	14<br>4.4 (2.4–7.3)	3<br>0.9 (0.2–2.7)	4<br>1.3 (0.3–3.2)
	60–69	313	5<br>1.6 (0.5–3.7)	8<br>2.6 (1.1–5.0)	2<br>0.6 (0.0–2.3)	4<br>1.3 (0.3–3.3)
	70–94	314	2<br>0.6 (0.0–2.3)	20**<br>6.4 (3.9–9.8)	3<br>1.0 (0.2–2.8)	9<br>2.9 (1.3–5.4)

Note: Data are expressed as number of seropositive individuals (n) and percentage (%) with exact 95% confidence intervals (CI) based on the binomial distribution (Clopper–Pearson method). Age groups are presented in years. Statistical significance of differences between age groups was assessed using the chi–square test for trend or Fisher's exact test, with the youngest age group (1–5 years) serving as the reference category. * — $p < 0.05$, ** — $p < 0.01$ indicate significantly higher seroprevalence compared to the reference group. A marked increase in IgG seropositivity to *B. burgdorferi* s.l. was observed in individuals aged 70 years and older in both regions, likely reflecting cumulative exposure over lifetime.

Table S3. Seroprevalence of *Borrelia* species in different administrative districts of St. Petersburg and Leningrad Oblast.

District	N	*B. burgdorferi* s.l			*B. miyamotoi*		
		IgM, n, % (95% CI)	IgG, n, % (95% CI)	IgM/IgG, n, % (95% CI)	IgM, n, % (95% CI)	IgG, n, % (95% CI)	IgM/IgG, n, % (95% CI)
St. Petersburg							
Vasileostrovsky	2	0<br>0 (0–100)	0<br>0 (0–100)	0<br>0 (0–100)	0<br>0 (0–100)	0<br>0 (0–100)	0<br>0 (0–100)
Vyborgsky	420	10<br>2.4 (1.1–4.4)	6<br>1.4 (0.5–3.1)	15<br>3.8 (2–5.9)	0<br>0 (0–0.9)	5<br>1.2 (0.4–2.8)	5<br>1.2 (0.4–2.8)
Kalininsky	4	1<br>25 (0.6–13.9)	1<br>25 (0.6–13.9)	1<br>25 (0.6–13.9)	0<br>0 (0–9.2)	0<br>0 (0–9.2)	0<br>0 (0–9.2)
Kirovsky	1	0<br>0 (0–100)	0<br>0 (0–100)	0<br>0 (0–100)	0<br>0 (0–100)	0<br>0 (0–100)	0<br>0 (0–100)
Kolpinsky	54	1<br>1.9 (0–10.3)	2<br>3.7 (0–13.4)	2<br>3.7 (0–13.4)	0<br>0 (0–6.3)	0<br>0 (0–6.3)	0<br>0 (0–6.3)
Krasnogvardeisky	214	5<br>2.4 (0.8–5.4)	6<br>2.8 (1.0–6.1)	10<br>4.7 (2.2–8.6)	0<br>0 (0–1.7)	5<br>2.3 (0.8–5.4)	5****<br>2.3 (0.8–5.4)
Krasnoselsky	138	5<br>3.6 (1.2–8.4)	5<br>3.6 (1.2–8.4)	8<br>5.8 (2.5–11.4)	0<br>0 (0–2.7)	3<br>2.2 (0.4–6.4)	3<br>2.2 (0.4–6.4)

Kronshtadtsky	10	0<br>0 (0–36.9)	1<br>10 (0.3–55.7)	1<br>10 (0.3–55.7)	0<br>0 (0–36.9)	0<br>0 (0–36.9)	0<br>0 (0–36.9)
Kurortny	31	0<br>0 (0–11.9)	1<br>3.2 (0–18.0)	1<br>3.2 (0–18.0)	0<br>0 (0–11.9)	0<br>0 (0–11.9)	0<br>0 (0–11.9)
Moskovsky	8	0<br>0 (0–46.1)	0<br>0 (0–46.1)	0<br>0 (0–46.1)	0<br>0 (0–46.1)	1<br>12.5 (0.3–69.7)	1<br>12.5 (0.3–69.7)
Nevsky	5	0<br>0 (0–73.8)	0<br>0 (0–73.8)	0<br>0 (0–73.8)	0<br>0 (0–73.8)	0<br>0 (0–73.8)	0<br>0 (0–73.8)
Petrogradsky	1	0<br>0 (0–100)	0<br>0 (0–100)	0<br>0 (0–100)	0<br>0 (0–100)	0<br>0 (0–100)	0<br>0 (0–100)
Petrodvortsovy	37	0<br>0 (0–10.0)	1<br>2.7 (0–15.0)	1<br>2.7 (0–15.0)	0<br>0 (0–10.0)	0<br>0 (0–10.0)	0<br>0 (0–10.0)
Primorsky	490	5<br>1.0 (0.3–2.4)	18<br>3.7 (2.2–5.8)	23<br>4.7 (2.8–7.0)	1<br>0.2 (0–1.1)	1<br>0.2 (0–1.1)	2<br>0.4 (0–1.5)
Pushkinsky	120	3<br>2.5 (0.5–7.3)	6<br>5.0 (1.8–10.9)	9<br>7.5 (3.4–14.2)	0<br>0 (0–3.1)	0<br>0 (0–3.1)	0<br>0 (0–3.1)
Centralny	1	0<br>0 (0–100)	0<br>0 (0–100)	0<br>0 (0–100)	0<br>0 (0–100)	0<br>0 (0–100)	0<br>0 (0–100)
Leningrad Oblast							
Boksitogorsky	134	4<br>3.0 (0.8–7.6)	9<br>6.7 (3.1–12.8)	13**<br>9.7 (5.2–16.6)	0<br>0.0 (0.0–2.8)	1<br>0.7 (0.0–4.2)	1<br>0.7 (0.0–4.2)
Volosovsky	140	2<br>1.4 (0.2–5.2)	5<br>3.6 (1.2–8.3)	7<br>5.0 (2.0–10.3)	2<br>1.4 (0.2–5.2)	2<br>1.4 (0.2–5.2)	4<br>2.9 (0.8–7.3)
Volkhovsky	120	0<br>0.0 (0.0–3.1)	2<br>1.7 (0.2–6.0)	2<br>1.7 (0.2–6.0)	1<br>0.8 (0.0–4.6)	1<br>0.8 (0.0–4.6)	2<br>1.7 (0.2–6.0)
Vsevolozhsky	139	2<br>1.4 (0.2–5.2)	5<br>3.6 (1.2–8.4)	7<br>5.0 (2.0–10.4)	1<br>0.7 (0.0–4.0)	1<br>0.7 (0.0–4.0)	2<br>1.4 (0.2–5.2)
Vyborgsky	141	2<br>1.4 (0.2–5.1)	3<br>2.1 (0.4–6.2)	5<br>3.5 (1.1–8.3)	1<br>0.7 (0.0–4.0)	0<br>0.0 (0.0–2.6)	1<br>0.7 (0.0–4.0)
Gatchinsky	131	1<br>0.8 (0.0–4.3)	3<br>2.3 (0.5–6.7)	4<br>3.1 (0.8–7.8)	1<br>0.8 (0.0–4.3)	1<br>0.8 (0.0–4.3)	2<br>1.5 (0.2–5.5)
Kingiseppsky	112	3<br>2.7 (0.6–7.8)	2<br>1.8 (0.2–6.5)	5<br>4.5 (1.5–10.4)	1<br>0.9 (0.0–5.0)	2<br>1.8 (0.2–6.5)	3<br>2.7 (0.6–7.8)
Kirishsky	138	2<br>1.4 (0.2–5.2)	3<br>2.2 (0.5–6.4)	5<br>3.6 (1.2–8.5)	2<br>1.4 (0.2–5.2)	2<br>1.4 (0.2–5.2)	3<br>2.2 (0.5–6.4)
Kirovsky	138	3<br>2.2 (0.5–6.4)	5<br>3.6 (1.2–8.5)	8<br>5.8 (2.5–11.4)	4<br>2.9 (0.8–7.4)	5<br>3.6 (1.2–8.5)	9***<br>6.5 (3.0–12.4)
Lomonosovsky	137	3<br>2.2 (0.5–6.4)	3<br>2.2 (0.5–6.4)	5<br>3.6 (1.2–8.5)	0<br>0.0 (0.0–2.7)	0<br>0.0 (0.0–2.7)	0<br>0.0 (0.0–2.7)
Lodeynopolsky	54	1<br>1.9 (0.0–10.3)	0<br>0.0 (0.0–6.8)	1<br>1.9 (0.0–10.3)	0<br>0.0 (0.0–6.8)	1<br>1.9 (0.0–10.3)	1<br>1.9 (0.0–10.3)
Luzhsky	139	1<br>0.7 (0.0–4.0)	6<br>4.3 (1.6–9.4)	7<br>5.0 (2.0–10.4)	0<br>0.0 (0.0–2.7)	5<br>3.6 (1.2–8.4)	5<br>3.6 (1.2–8.4)
Priozersky	139	1<br>0.7 (0.0–4.0)	3<br>2.2 (0.4–6.3)	4<br>2.9 (0.8–7.4)	0<br>0.0 (0.0–2.7)	2<br>1.4 (0.2–5.2)	2<br>1.4 (0.2–5.2)
Podporozhsky	125	6	7	13**	1	1	2

		4.8 (1.8–10.5)	5.6 (2.2–11.5)	10.4 (5.5–17.8)	0.8 (0.0–4.5)	0.8 (0.0–4.5)	1.6 (0.2–5.8)
Slantsevsky	128	4<br>3.1 (0.8–8.0)	4<br>3.1 (0.8–8.0)	7<br>5.5 (2.2–11.3)	1<br>0.8 (0.0–4.4)	2<br>1.6 (0.2–5.6)	3<br>2.3 (0.5–6.8)
Tikhvinsky	133	5<br>3.8 (1.2–8.8)	3<br>2.3 (0.5–6.6)	8<br>6.0 (2.6–11.9)	0<br>0.0 (0 .0–2.8)	1<br>0.8 (0.0–4.2)	1<br>0.8 (0.0–4.2)
Tosnensky	141	6<br>4.3 (1.6–9.3)	3<br>2.1 (0.4–6.2)	9<br>6.4 (2.9–12.1)	1<br>0.7 (0.0–4.0)	4<br>2.8 (0.8–7.3)	5<br>3.5 (1.1–8.3)

Note: Data are presented as number of positive cases (n) and percentage (%) with exact 95% confidence intervals (CI) calculated using the Pearson method. The "Total" column represents individuals positive for either IgM and/or IgG antibodies against the respective pathogen. Only districts with at least one positive case are shown in detail; full data are available upon request. Asterisks indicate districts with significantly elevated seroprevalence compared to the regional average: * — $p < 0.05$, ** — $p < 0.01$ (chi–square test with Yates' correction or Fisher's exact test where appropriate). Notably, Kirovsky district features the highest seroprevalence of *B. miyamotoi* (6.5%), while Boksitogorsky and Podporozhsky districts showed the highest exposure to *B. burgdorferi* s.l. (9.7% and 10.4%, respectively), suggesting localized foci of heightened transmission risk.
